# Longitudinal CTCs gene expression analysis on metastatic castration-resistant prostate cancer patients treated with docetaxel reveals new potential prognosis markers

**DOI:** 10.1007/s10585-021-10075-1

**Published:** 2021-02-26

**Authors:** Thais Pereira-Veiga, Miriam González-Conde, Luis León-Mateos, Roberto Piñeiro-Cid, Carmen Abuín, Laura Muinelo-Romay, Mónica Martínez-Fernández, Jenifer Brea Iglesias, Jorge García González, Urbano Anido, Santiago Aguín-Losada, Víctor Cebey, Clotilde Costa, Rafael López-López

**Affiliations:** 1 Roche-Chus Joint Unit, Translational Medical Oncology Group, Oncomet, Health Research Institute of Santiago de Compostela (IDIS), Travesía da Choupana s/n, 15706 Santiago de Compostela, Spain; 2 Present Address: Department of Tumor Biology, Center of Experimental Medicine, University Medical Center Hamburg-Eppendorf, 20246 Hamburg, Germany; 3 Medical Oncology Department, University Clinical Hospital of Santiago de Compostela, 15706 Santiago de Compostela, Spain; 4 Centro de Investigación Biomédica en Red Cáncer, CIBERONC, 28029 Madrid, Spain; 5 Liquid Biopsy Analysis Unit, Translational Medical Oncology Group, Health Research Institute of Santiago de Santiago de Compostela (IDIS), Travesía da Choupana s/n, 15706 Santiago de Compostela, Spain; 6 Genomes and Disease Lab. CIMUS, Universidade de Santiago de Compostela (USC), Avda. Barcelona 31, 15706 Santiago de Compostela, Spain; 7 Translational Medical Oncology Group (Oncomet), Health Research Institute of Santiago de Compostela (IDIS), 15706 Santiago de Compostela, Spain

**Keywords:** Prostate cancer, CRPC, CTCs, Liquid biopsy, Biomarkers, EMT, Taxanes

## Abstract

CTCs have extensively been used for the monitoring and characterization of metastatic prostate cancer, but their application in the clinic is still very scarce. Besides, the resistance mechanisms linked to prostate cancer treatment remain unclear. Liquid biopsies represent the most promising alternative due to the complexity of biopsying bone metastasis and the duration of the disease. We performed a prospective longitudinal study in CTCs from 20 castration-resistant prostate cancer patients treated with docetaxel. For that, we used CellSearch® technology and a custom gene expression panel with qRT-PCR using a CTCs negative enrichment approach. We found that CTCs showed a hybrid phenotype during the disease, where epithelial features were associated with the presence of ≥ 5 CTCs/7.5 mL of blood, while high relative expression of the gene *MYCL* was observed preferentially in the set of samples with < 5 CTCs/7.5 mL of blood. At baseline, patients whose CTCs had stem or hybrid features showed a later progression. After 1 cycle of docetaxel, high relative expression of *ZEB1* indicated worse outcome, while *KRT19* and *KLK3* high expression could predisposed the patients to a worse prognosis at clinical progression. In the present work we describe biomarkers with clinical relevance for the prediction of early response or resistance in castration-resistant prostate cancer patients. Besides, we question the utility of targeted isolated CTCs and the use of a limited number of markers to define the CTCs population.

**Supplementary Information:**

The online version contains supplementary material available at (10.1007/s10585-021-10075-1).

## Background

Prostate cancer (PC) is the fifth leading cause of cancer-related death worldwide and the second most commonly diagnosed cancer in men [1]. The incidence and mortality of PC correlate with increasing age and most of the mortality occurs after 65 years of age. Early detection and new advances in PC treatment are improving the survival rate and the quality of life of diagnosed patients; however, the prediction for the upcoming years is of 379,000 deaths worldwide in 2040 [1].

Androgen deprivation therapy (ADT) is the first-line treatment of choice for men with advanced PC. Eventually, the vast majority of these patients develops a disease progression known as castration-resistant PC (CRPC) [2]. The mechanisms involved in this process are still unclear, although it has been reported that continued signalling of androgen receptors (AR) is critical to the development of CRPC [3]. For advanced CRPC, taxanes, new anti-androgen inhibitors or Radium223 are active agents, but the development of biomarkers it is still needed to select the most effective and less toxic treatment for each patient [4].

Prostate-specific antigen (PSA) represents a reliable and useful biomarker for early detection and early diagnosis of disease progression and it has been reported that PSA screening has a potential benefit of decreasing deaths from PC in men aged 55 to 69 years old [5]. However, it does not give information about biological features of the disease and, for men above 70 years old, the data are less conclusive [5], losing its predictive role in metastatic CRPC (mCRPC) setting [6]. Since PSA is an androgen-dependent gene, its specificity could also be low, particularly when AR signalling-directed therapies are administered [7]. In this context, there is an urgent need to explore resistance mechanisms and biomarkers of prognosis as clinical tools for better patient management due to the absence of useful biomarkers and effective therapeutic options for the advanced PC patients.

Besides, patients with metastatic PC (mPC) usually exhibit a long evolution of the disease, therefore, the molecular information of the primary tumour, often does not reflect the current state of the disease at the molecular level. Furthermore, these patients usually develop bone metastases, which makes biopsy very difficult. Hence, liquid biopsy (LB) represents a promising alternative for the study of the metastatic disease in CRPC patients [8]. CTCs enumeration is the most extensively used approach since the CellSearch® System (Menarini Silicon Biosystems) is the only device that has achieved the level of regulatory clearance by the US Food and Drug Administration (FDA) [9–13]. Additionally, several studies and clinical trials have shown the utility of CTC analysis in patient outcome prediction, in disease monitoring during therapy, as response indicator, as well as endpoint biomarker or tumour phenotype reporter [14, 15].

Other publications showed the usefulness of CTCs as a source of tumour sample for the identification of predictive biomarkers. For example, AR splice variant 7 (ARv7) has been linked to resistance to enzalutamide or abiraterone [16–18]. Markou and colleagues have observed interesting changes in gene expression of CTCs during therapy [19] and it has been described a notable concordance among the expression of 9 genes in CTCs and paired spinal column metastasis reflecting that CTCs are an important source of information about the metastatic disease [20]. Other study has found a prognosis signature in CRPC patients CTCs [21]. However, most of the studies use an EpCAM-dependent approach for CTCs isolation, which does not consider all CTCs populations present in the bloodstream. This could lead to biased results and, if the methodology is not the same, data is not always comparable among studies. There are other factors that make it difficult to make comparisons between different studies, as different time points of sample analysis, different custom panels of genes for expression analysis assays or lack of consensus regarding normalization techniques.

Despite multiple publications in the field, so far, no biomarkers have been implemented for their use in the clinical routine. In this study, we aim to perform longitudinal CTCs enumeration by the EpCAM-based CellSearch® system together with a molecular profile characterization. As a novelty, we have used an EPCAM-independent negative enrichment approach for CTCs isolation, the inclusion of heterogeneity on the selected panel of genes and, the pairing peripheral blood mononuclear cells (PBMCS) expression as a normalizer for each patient sample.

## Methods

### Patient characteristics

20 patients diagnosed with mCRPC at the Clinical Hospital of Santiago de Compostela (Spain) were enrolled in the study between October 2016 and July 2019. This study was approved by the local ethics committee (Ethics Committee of Galicia approval reference number 2015/772) and all patients gave written informed consent**.**

Inclusion criteria were: histologically confirmed prostate adenocarcinoma, stage IV and candidate to receive treatment with chemotherapy; age ≥ 18 years; PS-ECOG 0–2 and proper function of organs to receive antineoplastic treatment. The median age of the patients was 74.6 years (range 59–86 years). In total, 90% of the patients had received among 1 to 3 previous hormone treatments before the development of metastasis. All the patients had bone metastasis and 65% of them showed metastasis in additional sites. All the patients enrolled in this study were treated with docetaxel and in 19 out of 20 the disease progression took place within the study; 15 patients died by mCRPC with a median time of 392 days (range 159–951) and 2 patients died by a different cause: one by respiratory infection and heart failure, with no evidence of progression and other by intestinal perforation. Clinical data of this mCRPC cohort is summed in Table S1.

### Clinical samples and CTCs analysis

Two EDTA-coated vacutainer tubes (Becton Dickinson) of 7.5 ml of peripheral blood and one additional 10 mL CellSave Preservative tube (Menarini-Silicon Biosystems) were collected per patient at different time points: 20 samples before the start of systemic therapy (visit 1, V1), 20 samples after 1 cycle of chemotherapy (visit 2, V2), and 13 samples after clinical progression (visit 3, V3) determined by CT-Scan. Blood samples were processed within two hours after withdrawal. All samples were anonymised and encoded before the analysis.

In total, 53 samples were included in this study. Blood samples were analysed in parallel with the label-independent antibody cocktail RosetteSep™ CTC Enrichment Cocktail Containing Anti-CD56 (STEMCELL Technologies) (V1, n = 20; V2, n = 20 and V3, n = 13) and with the EpCAM-based CellSearch® System (Menarini Silicon Biosystems) as previously described [22] (V1, n = 19; V2, n = 20 and V3, n = 13).

In addition, the PBMCs of the patients were isolated from one 7.5 mL EDTA tube of peripheral blood by density gradient centrifugation protocol (Lymphoprep™, STEMCELL Technologies) in SepMate™ tubes (STEMCELL Technologies) according to manufacturer’s instructions.

Enriched cells isolated with RosetteSep™ and PBMCs were placed in RNAlater™ Solution (Invitrogen, ThermoFisher Scientific) and kept at -80ºC until further analyses.

### Gene expression analysis

Extraction of RNA from the enriched tumour cells and the PBMCs fraction was performed using AllPrep DNA/RNA Mini Kit (Qiagen) following the manufacturer’s protocol. 11 µL of total RNA were retrotranscribed into cDNA with SuperScript III (ThermoFisher Scientific). Samples were preamplified with Taqman Preamp Master Mix (ThermoFisher Scientific) due to the low recovery of RNA in the enriched tumour cell samples. cDNA expression was analysed on a LightCycler 480 II (Roche Diagnostics) with TaqMan Gene Expression Master Mix and TaqMan probes (Applied Biosystems) for a custom panel of 20 genes (Table S2). *B2M* was used as a reference gene and after data normalization, gene expression from CTCs was relativized to the autologous PBMCs transcripts and relative expression > 1.5 fold was considered as high relative expression in CTCs. Not detection or < 1.5 fold was considered as negative CTCs expression. In order to clarify the visualization of the heatmap, relative expression was codified from − 1 to 4 points. Thus, no detection was 0 (light blue in the heatmap), relative expression < 1.5 was − 1 (white); relative expression > 1.5–5 was 1; > 5–20 was 2; > 20–100 was 3 and > 100 was 4 (blue sequential intensity).

### Statistical analysis

Statistical analysis was performed using GraphPad Prism 6.01 software (GraphPad Software Inc.) and R Studio Version R-3.6.3. ClustVis tool was used for heatmap performance. Shapiro–Wilk Test was used to check for normality distribution. The Wilcoxon signed-rank test was used to compare CTCs longitudinal enumeration and for gene expression comparison among visits. Assuming PBMCs contamination in the enriched fraction of CTCs, the expression of the autologous PBMCs was used as a normalizer. Fisher test and Chi-square test were used to study the association between gene expression of the CTCs and with enumeration data. Dependency analysis between gene expression and clinical data were tested by Fisher´s exact test. Progression free survival (PFS) and overall survival (OS) were visualized using Kaplan–Meier plots and tested by the log-rank test. Only p values < 0.05 were considered statistically significant.

### Pan-Cancer Atlas 2018 dataset analysis

RNA-Seq publicly available data from TCGA Pan-Cancer Atlas 2018 project has been downloaded from cBioportal repository [23–25]. Expression has been batch normalized from Illumina HiSeq RNASeqV2 and quantified by RSEM method (RNA-Seq by Expectation–Maximization) [26]. *MYCL* expression median has been calculated across this dataset, classifying patients in two groups: “high *MYCL*” if their expression is higher than the median or “low *MYCL*” if not. Kaplan Meier analysis has been performed to assess, using a log-rank test, whether there were differences for PFS probability among these groups.

## Results

### CTCs enumeration by CellSearch

**Fig. 1:**
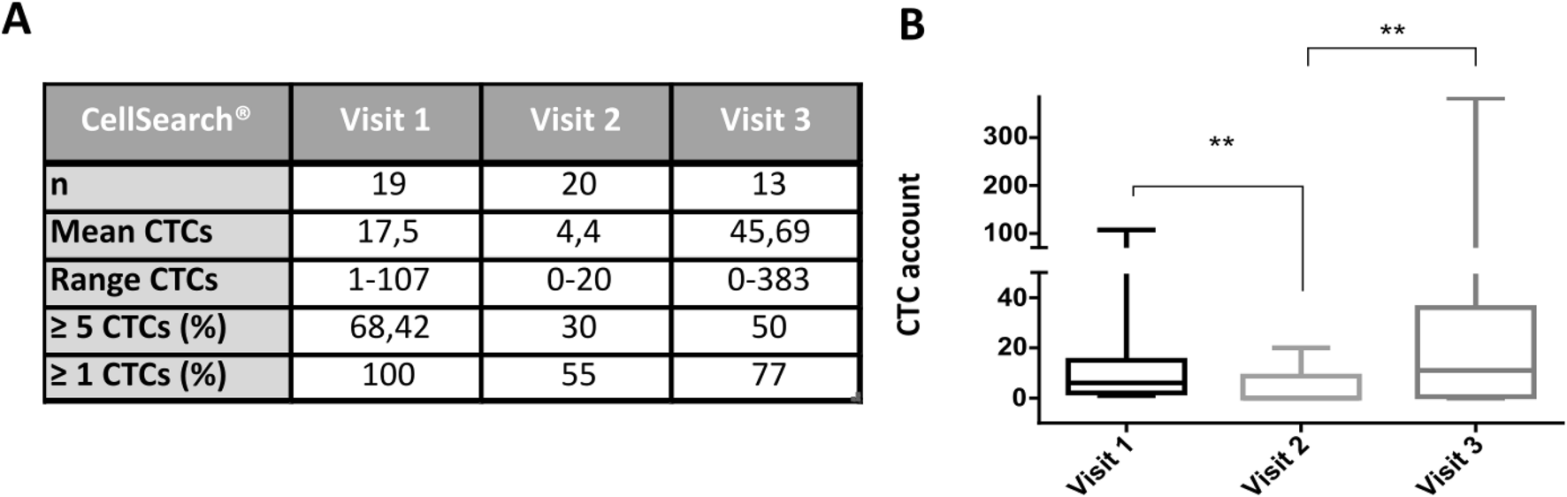
**a** Summary table of CellSearch® data at three different time points. **b** Longitudinal CTCs enumeration by CellSearch® on the mCRPC patient cohort. P-values were calculated using the Wilcoxon test (** < 0.001) due to the lack of normality in the data

The Cellsearch is an EpCAM-based system that only considers those CTCs with epithelial features (Figure S1). CTCs were isolated and enumerated with the CellSearch® system in 20 mCRPC patients in three different time points: before chemotherapy (Visit 1, V1), after one cycle of docetaxel therapy (Visit 2, V2) and at radiological progression determined by CT-Scan (Visit 3, V3). At V1, 1 sample (#UM164) was not evaluated due to insufficient amount of blood. Among the 19 patients analysed at V1, 13 patients (68.5%) had ≥ 5 CTCs per 7.5 mL blood and 6 patients (31.5%) had 1–4 CTCs. After 1 cycle of docetaxel (V2), we observed a decrease in the CTCs counts in 84.2% of the patients (p = 0.002). Thus, 6 patients (30%) had ≥ 5 CTCs and 3 patients (15%) had 1–4 CTCs, while in 11 patients (55%) no CTCs were identified. At clinical progression (V3), among the 13 patients analysed, we observed an increase in the CTCs counts when compared with V2 (p = 0.006): 7 patients (54%) showed an increase in the CTCs numbers and had ≥ 5 CTCs; and 3 out of 7 switched from good prognostic to the bad prognostic group; 3 patients (23%) had 1–4 CTCs and no CTCs were detected in 3 patients (23%) (Fig. 1).

We identified significant differences between samples with < 5 and ≥ 5 CTCs/7.5 mL of blood being high CTCs account data associated with high PSA levels and receiving ≤ 2 previous hormone treatments. There is an imbalance in the number of cases previously treated with ≥ 2 hormone-deprivation therapy that may be biasing the result. Other clinical data as age, Gleason score, the type of castration, LDH or ALK-P levels did not show a statistical difference between having < 5 CTCs or not (Table 1).

**Table 1:** Associations between CTCs number and the clinical-pathologic characteristics of the cohort of patients at the diagnostic of metastasis

Category	N	%	< 5CTCs	≥ 5 CTCs	p value
Age					
< 75 years	10	52.6	5	5	0.14
≥ 75 years	9	47.3	1	8	
GLEASON score					
≤ 7	10	52.6	1	9	0.05
> 7	9	47.3	5	4	
Type of castration					
No surgery	15	78.9	4	11	0.55
Surgery	4	21	2	2	
Prior hormone therapy					
≤ 2	14	73.6	2	12	0.01
> 2	5	26.3	4	1	
Visceral metastasis					
No	13	68.42	4	9	1
Yes	6	31.58	2	4	
LDH_basal					
Low	8	50	3	5	0.56
High	8	50	1	7	
ALK-P_basal					
Low	9	47.3	5	4	0.05
High	10	52.6	1	9	
PSA_basal					
Low	10	52.6	6	4	0.01
High	9	47.3	0	9	

P-values were calculated using the Fisher exact test. For LDH, ALK-P and PSA levels, the median value of the analysed cohort was considered as a threshold for low or high category (see Table S1).

Next, we analysed if CTCs count was able to predict patient´s outcome as previously described in CRPC. To address this aim, a survival analysis was performed to evaluate OS and PFS in relation to CellSearch® data, considering the previously defined good prognosis (≥ 5 CTCs) or bad prognosis (< 5 CTCs) cut off. Patients with ≥ 5 CTCs tended to a shorter OS and PFS both at V1 (PFS, p = 0.16; OS, p = 0.6, log-rank test), V2 (PFS, p = 0.2; OS, p = 0.05, log-rank test) and V3 (OS, p = 0.09, log-rank test) compared with patients with < 5 CTCs, however statistical significance was not reached (Figure S2).

Taken together, these analyses indicate that the number of EpCAM + CTCs is reduced after chemotherapy and it increases again at clinical progression but it did not predict outcome in this patient cohort.

### Gene expression profile in the CTCs enriched fraction

In parallel to the CellSearch® enumeration, we performed a gene expression analysis on CTCs isolated with a negative enrichment approach in 20 patients (n, V1 = 20, V2 = 20, V3 = 13). We analysed the expression of a gene panel in the CTCs enriched fraction in relation to the expression found in the paired PBMCs’ fraction.

CTC-positive samples were defined as those with at least one epithelial (*CDH1*, *EpCAM* or *KRT19*), one mesenchymal (*SNAI1*, *VIM* or *ZEB1*) or one stem (*ALDH1A1* or *PROM1*) cell marker with higher expression regarding to the paired PBMCs expression (ΔΔct ≥ 1.5). Only one patient (#UM30) was considered CTC-negative both at V1 and V2 following this criterion which agrees with CellSearch® data (V1 = 3 CTCs; V2 = 0 CTCs). CTC-positive samples (n = 51) exhibited different frequency of expression for the epithelial (*CDH1*: 88.2%, *EpCAM*: 45.1%, *KTR19*: 45.1%), mesenchymal (*SNAI1*: 58.8%, *VIM*: 45.1% or *ZEB1*: 41.1%) and stem cell markers (*ALDH1A1*: 41.1%, *PROM1*: 45.1%). Interestingly, the CTC-positive samples showed a very heterogeneous pattern of expression, displaying a hybrid epithelial-mesenchymal phenotype in most of the analysed samples (70.6%) while 21.6% were exclusively epithelial and 7.8% expressed only mesenchymal markers. Regarding the other analysed genes, we observed variable expression among the samples (in percentages of CTCs positive samples): for the cell cycle-associated genes, we observed an increased relative expression of *CCND1* (56.8%), *CDK4* (50.9%), *E2F4* (78.4%) and *RB1* (68.6%). The PC related gene *KLK3* was expressed in more than half the samples (54.9%). *BCL2* and the proto-oncogene *MYC* showed lower relative expression in 90.2% and 92.1% respectively, although *MYCL* had higher relative expression in 64.7% of them. In addition, *CTNNB1* and *GDF15* showed also higher expression in 62.7% and 78.4% of the samples, respectively.

**Fig. 2:**
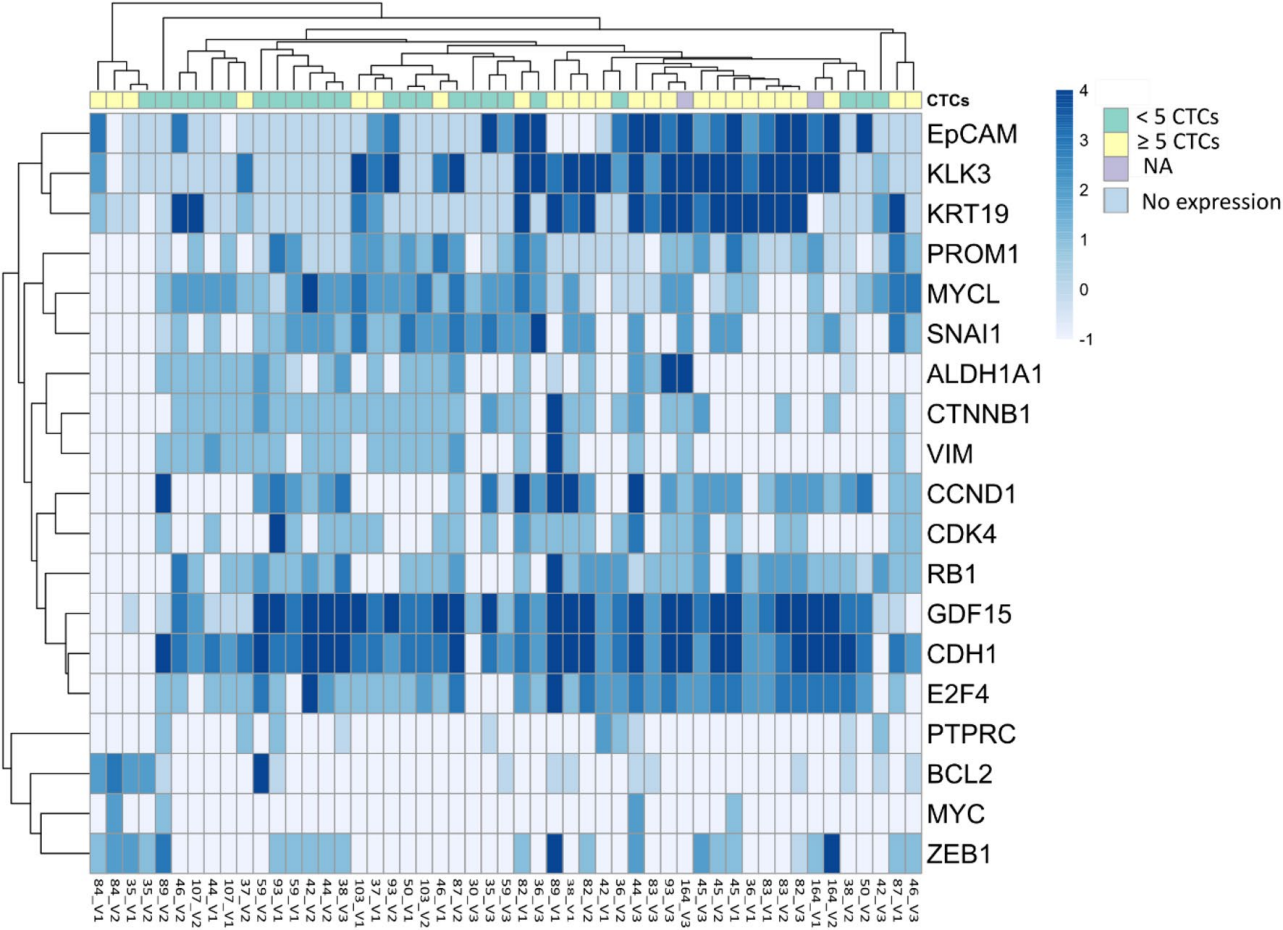
Clustered heatmap depicting CTCs gene expression levels and CTCs account (by CellSearch®) for all the analysed samples (Visit 1 to 3) (NA: not available). Green: < 5 CTCs; Yellow: ≥ 5 CTCs; Purple: NA; Light blue indicates no expression in CTCs

Next, we studied the association between gene expression and the number of CTCs (CellSearch® data) for each sample. For that, we consider a high expression as ≥ 1.5 fold change. We identified differential gene expression profiles depending on whether the samples had ≥ 5 or < 5 CTCs. Patients with ≥ 5 CTCs had a higher relative expression of the epithelial marker *KRT19* or the prostate-specific marker *KLK3* (p < 0.0001) while EpCAM higher expression was near significance (p = 0.057) (Fig. 2) and *KLK3*, *KRT19* and *EpCAM* relative expression associated also among them (p < 0.01). Interestingly, *MYCL* relative expression was associated with the good prognosis group (< 5 CTCs determined by CellSearch®) (p = 0.004) and also with high relative expression of stemness markers (*ALDH1A1* and *PROM1*) and *CTNNB1*, as well as mesenchymal markers as *SNAI1* and *VIM* but inversely to *ZEB1*. All the other markers were expressed independently of the CTCs counts and, remarkably, *CDH1* and *GDF15* are widely expressed in a high percentage of the samples (Fig. 2).

CDH1, GDF15, RB1, E2F4, MYCL and CTNNB1 genes showed increased relative gene expression in CTCs in most of the patients during the three visits, while in visit 3 the majority of the patients showed EpCAM, CCND1 or CDK4.

We next analysed if the absolute relative expression was different between patients having < 5 CTCs or those having ≥ 5 CTCs. We found that E2F4, KLK3 and KRT19 were statically different between both groups, showing higher values in the poor prognosis one.

Lastly, we studied the relationship between the clinic-pathological characteristics of the patients’ and their gene expression data. We found that CTC-positive samples by gene expression analysis from patients with low Gleason score showed a higher relative expression of *CCND1* (p = 0.019) and *RB1* (p = 0.004) at V1. No other association was found with clinical data.

Hence, these results suggest that CTCs in circulation display a hybrid epithelial-mesenchymal phenotype, showing also high expression of cell cycle regulation and PC-related genes. Although there is an association of some of the analysed genes with CTCs numbers, the expression profile of the CTCs did not group the samples by visits or patients except for sporadic cases.

### CTCs gene expression and its prognostic value

In order to determine if the gene expression of CTCs of the analysed markers had prognostic value, we applied the Cox regression for each of the visits independently, considering only CTC-positive samples (by our gene expression approach criterion). We defined 2 groups depending on CTCs expression value relative to the PBMCs as high or low. Overall, the mean follow-up period was 467 days, 19 out of 20 patients eventually developed progression and 15 out of 20 died during the follow-up.

**Fig. 3:**
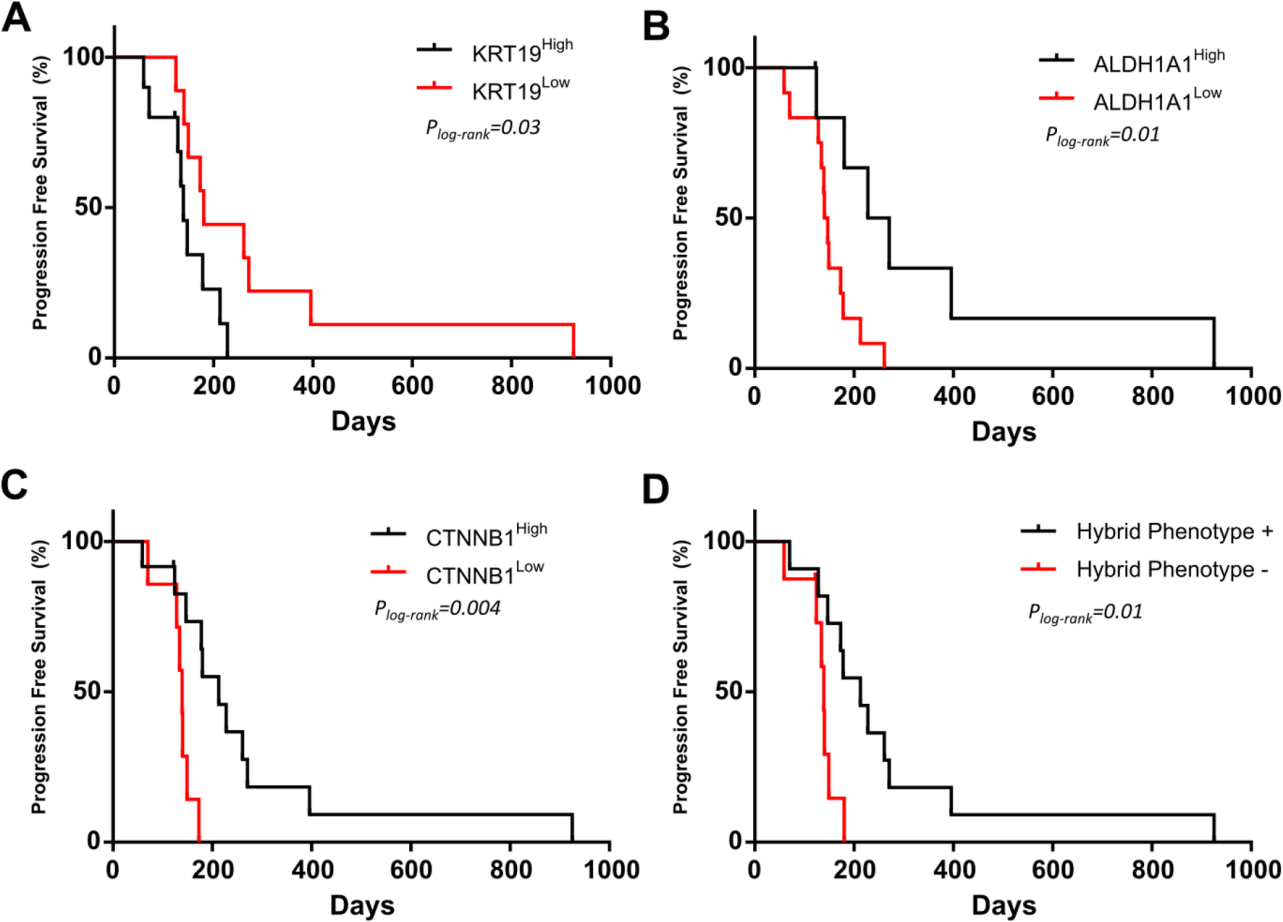
Kaplan–Meier plots for PFS at V1 based on the expression of **a** *KRT19*; **b** *ALDH1A1*; **c** *CTNNB1*; **d** hybrid phenotype which means expressing epithelial, mesenchymal and stem markers. P-values were calculated using the log-rank test

Of note, at V1, those patients with CTCs displaying epithelial characteristics had shorter PFS. Thus, a high expression of *KRT19* (p = 0.03, log-rank test, 139 vs 180 days) (Fig. 3a) predicted a worse prognosis. Furthermore, *EpCAM* showed the same trend (Table S3). In contrast, patients whose CTCs showed a higher relative expression of stem markers, *CTNNB1*, or those CTCs with a hybrid phenotype (that includes epithelial, mesenchymal and stem cell characteristics) showed a later disease progression (Fig. 3b–d and Table S3). In addition, high *MYCL* also tended to predict better outcome (PFS, p = 0.07; OS, p = 0.06, log-rank test) (Table S3).

**Fig. 4:**
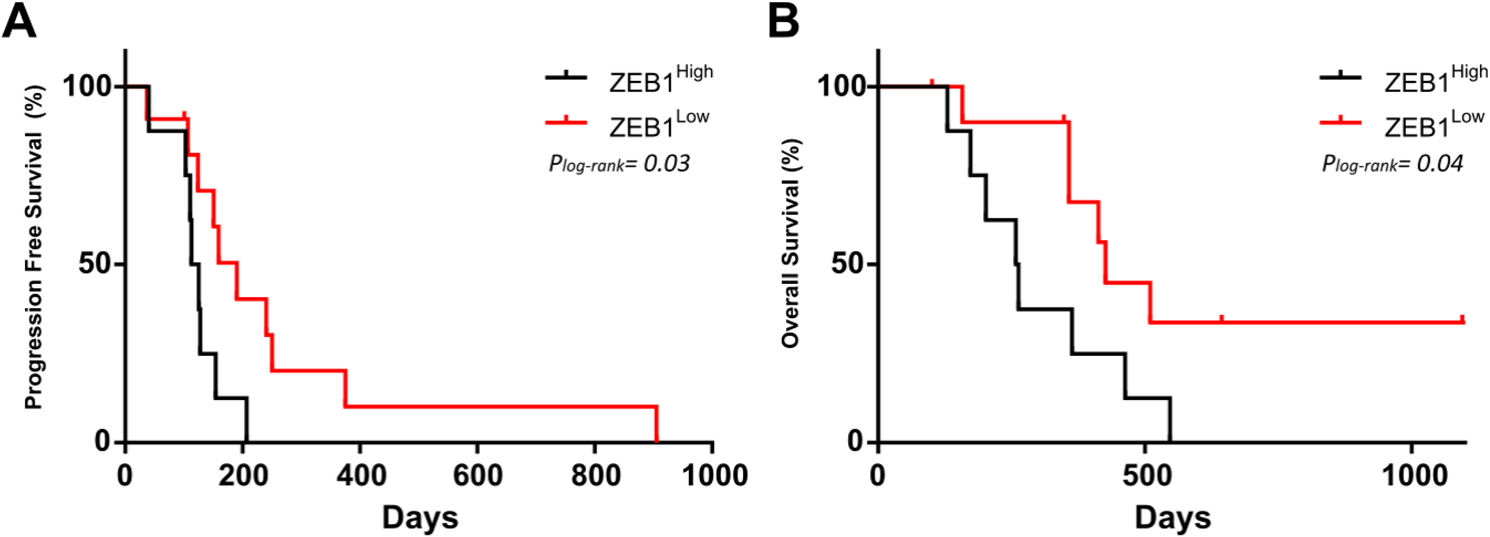
Kaplan–Meier plots based on the relative expression of *ZEB1* gene expression for PFS (**a**) and OS (**b**). P-values were calculated using the log-rank test

To evaluate early response to therapy, CTCs were analysed after 1 cycle of docetaxel. Thus, patients with CTCs with high expression of *ZEB1* in V2 had both shorter PFS (p = 0.03, 119 vs 190 days, log-rank test) and OS (p = 0.04, 260 vs 426 days, log-rank test) (Fig. 4a, b). In addition, CTCs with high expression of *CDK4* were also linked to poorer outcome (PFS, p = 0.03, log-rank test) (Table S3).

**Fig. 5:**
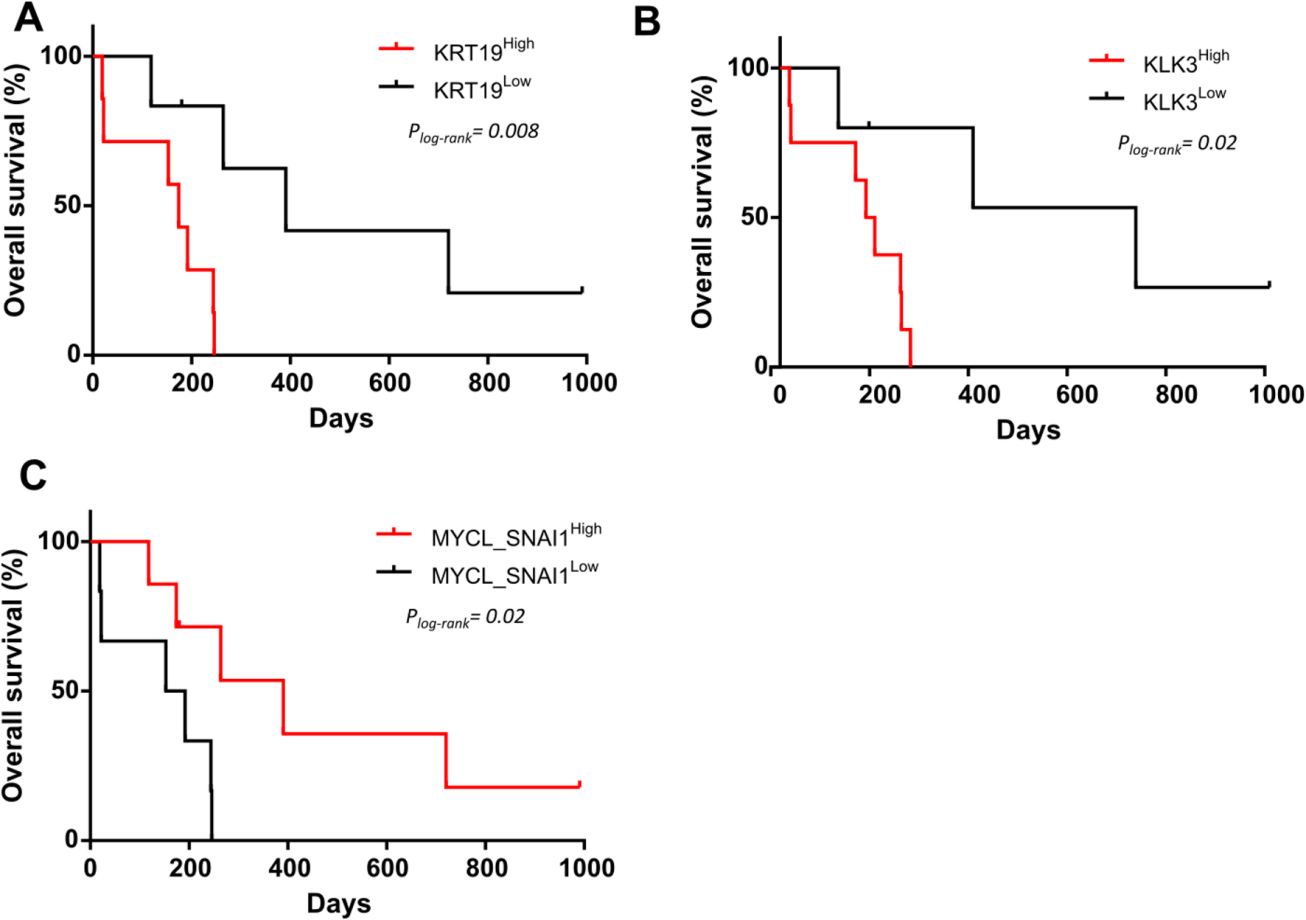
Kaplan–Meier plots for OS at V3 based on the expression of **a** *KRT19*; **b** *KLK3*; **c** *MYCL*^high^*SNAI1*^high^. P-values were calculated using the log-rank test

Lastly, we studied variations in CTCs gene expression at the clinical progression to identify if their molecular profile could predict the patients’ outcome in terms of OS. Indeed, at V3, high relative expression of either *KRT19* or *KLK3* in the CTCs enriched fraction was able to predict shorter OS (p = 0.008, 174 vs 391 days, log-rank test and p = 0.02, 183 vs 720 days, log-rank test, respectively) (Fig. 5a, b).

Since high *MYCL* and high *SNAI1* expression was associated with low expression of *KRT19* (p = 0.02, Fisher´s Exact test) at V3, we next explored whether the high expression of both *MYCL* and *SNAI1* was able to predict clinical outcome. We found that patients with high expression of these genes had a mean increase of 219 days in OS (p = 0.02, log-rank test) (Fig. 5Cc) and that *MYCL*^high^*SNAI1*^high^*KRT19*^low^ gene signature predicts OS with a superior statistical power (p = 0.008, log-rank test).

Next, we performed an additional analysis to check the prognostic value of the proposed markers regardless of the time. Since all patients’ present disease progression throughout the follow-up, we have differentiated those that do it early and late, considering median progression (days). At V1, high expression of *ALDH1A1* or *CTNNB1* associated with late progression (Fisher test, p = 0.04 and p = 0.004 respectively). After docetaxel therapy (V2), high *ZEB1* expression was observed mainly in those patients with early tumour progression (Fisher test, p = 0.02) or death (Fisher test, p = 0.04). For the other suggested biomarkers, there is a similar trend in the data. In this way, low *MYCL* expression or the lack of hybrid phenotype in V1 associated with early progression, although did not reach statistical significance.

As a summary, the expression analysis of CTCs in mCRPC patients allowed us to identify prognostic biomarkers in different collecting time points of the disease.

### High MYCL expression has prognostic value in localized PC patients

**Fig. 6:**
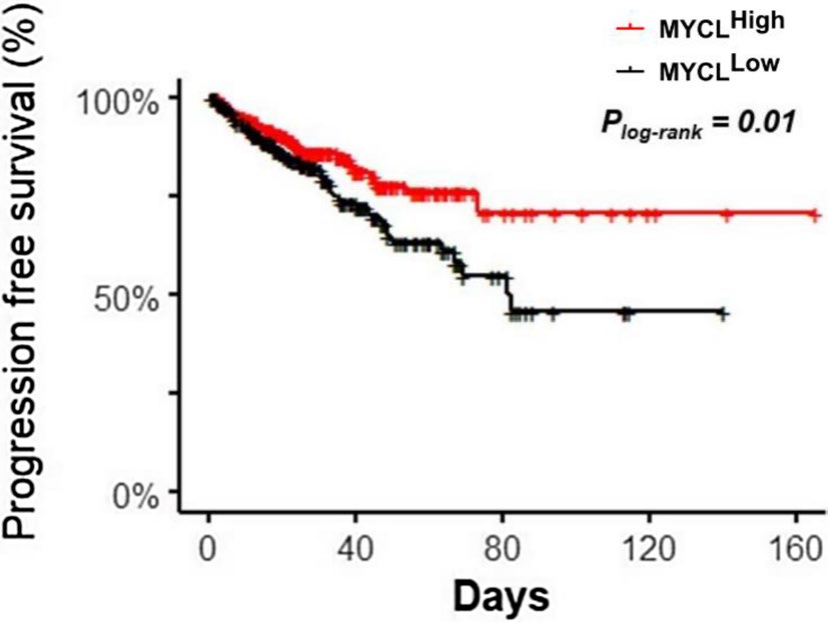
Kaplan–Meier plot for PFS of *MYCL* gene expression on primary tumour tissue samples from 492 PC patients with localised disease (TCGA Pan-Cancer Atlas 2018 database). P-value was calculated using the log-rank test

Since there is no *MYCL* gene expression reported data on primary tumour or CTCs from PC patients we explored the public available databases in order to study *MYCL* status. We focused on TCGA Pan-Cancer Atlas 2018 dataset (see Methods), which includes RNA-Seq data for 492 PC patients with localised disease, observing that *MYCL* is expressed in primary prostate tumours. As 485 out of 492 patients survived during the follow-up time while 93 patients progressed, we focused our analysis on PFS instead of on OS. We detected that those patients with an expression of *MYCL* higher than its median expression across the whole dataset showed a longer PFS and lesser progression-free probabilities (Fig. 6).

## Discussion

In regard with the study and analysis of CTCs, its enumeration has been widely linked with patients’ outcome [9, 12, 27] and, recently, used to assess treatment efficacy in mCRPC patients [11, 15]. However, CTC enumeration can only inform on disease progression. Besides account, the molecular characterization of CTCs has been proposed as a valuable source of predictive biomarkers [18, 28]. However, in mCRPC patients, there are neither markers for effective therapy selection nor markers with prognostic value for clinical use.

In the present work, we performed a longitudinal analysis of the CTCs enumeration and phenotypic characteristics, before and after chemotherapy. We aimed to decipher the CTCs phenotype during the disease and to study the relevance of CTCs analysis as indicators of therapy efficacy and prognostic biomarkers. In this mCRPC cohort, we observed a decrease in the number of CTCs after therapy and an increase in the clinical progression. CTCs enumeration was not enough to predict patients’ outcome except for OS after therapy, where the presence of ≥ 5 was in the limit of significance (pre-defined cut-off). Other clinical parameters as PSA, 50% decline or PSA level (measured at V1) did not have prognostic value in this set of patients (data not shown). Nevertheless, we found higher PSA levels in the bad prognostic patients’ group (≥ 5 CTCs by CellSearch®). The PSA level and PSA kinetics have been used as predictive indicators of disease progression and are the most widely used biomarkers for PC follow-up, however, there is some controversy regarding its prognostic impact on survival [29]. These disagreements found in our work with previous studies were probably due to the small size of our cohort.

To study the phenotypic characteristics of the CTCs at the different time points, we analysed the gene expression of a negative enriched CTCs fraction. This kind of enrichment approach avoids the bias of epitope-dependent methods allowing the analysis of a wider CTCs phenotype spectrum. In line with previous studies, CTCs gene expression analysis revealed the existence of a hybrid CTCs population with epithelial, mesenchymal and stem features [19]. As CTCs were analysed in bulk there is no way to know if a single CTC is co-expressing different markers or if within the same sample some CTCs are on an epithelial state and some in a mesenchymal or stem one. Both approaches have been supported by previous studies and mixed CTCs phenotypes have been reported [30–32]. Additionally, different metastasis sites could be shedding CTCs into the bloodstream leading to different CTCs phenotypes.

In this study, the presence of ≥ 5 CTCs/7.5 mL of blood determined by CellSearch® (EpCAM +) was associated with the expression of the epithelial markers *KRT19* and *EpCAM*, this latter close to statistical significance. In addition, samples with ≥ 5 CTCs were associated with the high relative expression of the prostate-specific gene *KLK3*, which also associates with epithelial features (*KRT19* and *EpCAM* expression). *KLK3* widely expressed in normal prostate epithelium. However, a loss in this protein expression has been reported in undifferentiated prostate carcinomas [33], which could explain a lack of *KLK3* expression in some CTCs sets. Given that *KLK3* expression is associated with epithelial markers, isolation approaches that consider only EpCAM + populations could lead to biased results towards *KLK3* expression.

Interestingly, we identified other genes with higher relative expression in CTCs samples regardless of their CTCs count. Thus, *CDH1*, *GDF15*, *CTNNB1*, *RB1* or *E2F4* were expressed in more than 60% of the samples. Remarkably, we found that CTCs expressing high *MYCL* were preferentially found in the CellSearch® good prognosis group (< 5 CTCs) and its expression was associated with both stem (*ALDH1A1* and *PROM1*) and mesenchymal markers (*SNAI1* and *VIM*, but no with *ZEB1*). Other genes as *MYC* or *BCL2* were less expressed in CTCs than in PBMCs, reinforcing the importance of considering the paired PBMCs expression for CTCs gene expression analysis. In addition, our data highlights the relevance of unbiased CTCs isolation methods and reflects the appropriateness of this negative enrichment approach. In this regard, few studies have included paired PBMCs data but it has been proved that PBMCs expression bias CTCs expression and should be included in the analysis [34].

One novelty of this work is the analysis of serial samples that allows the study of therapy impact on CTCs expression profile. Thus, at baseline, patients having CTCs with epithelial features show a tendency towards a worse outcome (PFS). This is in agreement with previous reports which described that only the *EpCAM* high population had an impact on the outcome of the patients when comparing *EpCAM* high and *EpCAM* low CTCs [35]. On the contrary, a stem or hybrid phenotype indicates a larger time to progression. The expression of stem markers or a hybrid phenotype has been linked to resistance to therapy and worse outcome in solid tumours [36]. Our CTCs data point in the opposite direction and agree with Scher and colleagues that suggest that patients with high heterogeneity scores would likely survive longer on taxanes than ADT therapy [30].

In accord with the previously mentioned association of *MYCL* expression to the good prognosis group (< 5 CTCs by CellSearch®), higher relative expression of *MYCL* on CTCs at diagnose of metastasis may be related to a larger time until progression. In this regard, it has been reported that *MYC* could be a decisive factor in the fate of mitotic cells driving the expression of an apoptotic network that initially sensitizes PC cells to antimitotic drugs such as taxanes, as reported by Topham and colleagues in breast, ovarian, lung and colon cancer cells [37]. *MYC* has been defined as a tumour driver in PC and has been described to be overexpressed in the earliest phases of the disease, being a key precursor lesion to invasive prostatic adenocarcinoma [38]. In CTCs, little is known regarding *MYC* expression and its prognostic role has not been described [39] but our data point to *MYCL* in detriment of *MYC* as a putative biomarker in mCRPC.

Although differential expressions have been described in different tumour types, the MYC family proteins (MYC, MYCN and MYCL) have highly conserved domains, which suggests that the mechanisms through which they carry out tumorigenesis are similar. MYCL has shown a sturdier and more specific activity in the generation of induced pluripotent stem cells (iPSC) in vitro compared to MYC [40]. Besides, the molecular mechanisms responsible for iPSC generation and tumorigenesis could overlap to a great extent [40]. MYCL appears frequently overexpressed in small cell lung cancer, but it is still the least explored member of the MYC family. Thus, to the best of our knowledge, there is only one publication reporting MYCL in localized PC [41], where they identified and validated a new recurring *MYCL* amplification, which is associated with *TP53* deletion and unique profiles of DNA damage and transcriptional dysregulation. In this regard, we have seen that *MYCL* is also highly expressed in PC localized tumours (Pan-Cancer database) [23] (data not shown). Having high *MYCL* expression shows the same trend as seen in CTCs, supporting the potential of *MYCL* as a prognostic biomarker of larger PFS or transient clinical response.

In this study, we also aimed to evaluate an early response, analysing CTCs after just one cycle of docetaxel, while the conventional response assessment is performed by imaging after 3 cycles of treatment. Of note, the survival analysis after therapy leads to remarkable data never described so far. Hence, high *ZEB1* expression also associates with early disease progression reinforcing the survival test data and suggesting a prognostic value for both PFS and OS. It has been previously described that epithelial-mesenchymal transition (EMT) confers therapeutic resistance to docetaxel, invasive properties and has a negative impact on the survival of PC patients [42–45]. This has been demonstrated in tissue from primary tumours and metastasis, as well as in PC cell lines [46]. Interestingly, we found a non-redundant role of *ZEB1* and *SNAI1* and a very limited role for *VIM*. This finding highlights the importance of considering different markers corresponding to the same phenotype since they could be showing different patterns of spatial–temporal expression in both physiological and pathological conditions, and could be controlled by different upstream signals [47–49].

It is usually assumed that EMT induction entails a downregulation of *CDH1*. Despite different studies in CRPC patients linking an induction in EMT markers with the transcriptional repression of *CDH1*, in CTCs we found both *CDH1* and EMT markers. This is in accordance with Hanrahan and colleagues that described that DU145 docetaxel-resistant cells maintained high *CDH1* expression regardless of an up-regulation of *ZEB1* and *ZEB2* [43]. Furthermore, in a xenograft model of bone metastasis, Putzke et al. observed that epithelial cells injected in the mouse tibia were able to growth while mesenchymal cells were not, and, additionally, *ZEB1* and *CDH1* were co-expressed in this mouse model [50]. Besides, *CDH1* extensive expression could be evidencing the role of *CDH1* as a survival factor and metastatic promoter as described by Padmanaban and colleagues in breast cancer models [51].

Those CTCs with mesenchymal features could gain invasion capabilities and presumable be responsible for the formation of new metastatic sites and hence disease progression. At clinical progression, we found that *KRT19* or *KLK3* expressing CTCs predicts shorter OS and EpCAM was also expressed in most of the analysed patients at V3. In addition, we also found that having CTCs with *MYCL*^high^*SNAI1*^high^ expression is linked with longer survival time in this patient´s cohort.

**Fig. 7:**
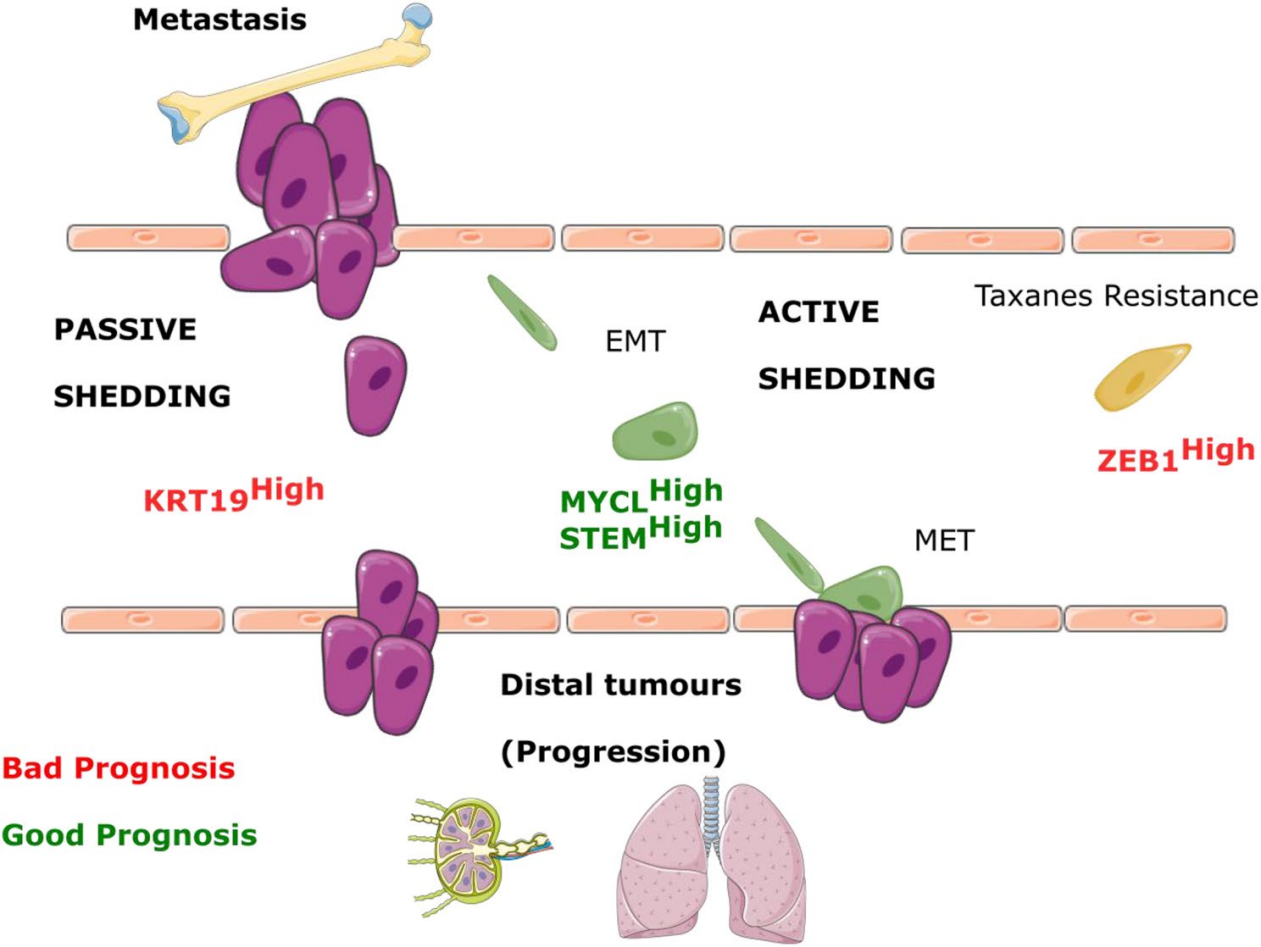
Hypothetical scheme illustrating how CTCs phenotypes could represent different alternatives of metastasis. Epithelial CTCs may represent a passive shedding and could be indicating a higher tumour burden. Stem cells, MYCL high expression or CTCs with hybrid phenotype would develop an active shedding which implies different aspects of the metastatic cascade as EMT. Thus, at V1 (progression to hormonal treatment), two possible outcomes of worse (red) or better (green) prognosis could happen. After one cycle of docetaxel, high relative expression of *ZEB1* predicts taxanes resistance and a worse outcome. *MYCL* may be controlling the hybrid phenotype and cancer stem cell potential that can undergo through EMT process before tumour re-growth

The prognosis value of the epithelial markers could indicate the presence of a higher number of epithelial CTCs in the bloodstream. They could be representing a higher tumour burden with passive tumour cells shedding. On the contrary, MYCL could be switching to a mesenchymal genetic program that can drive lineage plasticity and phenotypic heterogeneity, characteristic of some cancer types [52]. The stem or mesenchymal phenotype could represent a tumour population with active shedding. The active shedding includes intravasation, extravasation and EMT/MET process in distal organs. This hypothesis would explain the later progressions found in this latter phenotype (Fig. 7).

On the other side, to the best of our knowledge, there is no prognostic value reported in CTCs for *CDK4* but Gulappa and colleagues suggested that the modulation of *CDK4* expression could be an attractive target in the treatment of CRPC [53] such as happens in breast tumours [54]. Therefore, CTCs analysis could act as a surrogate-monitoring tool for the follow-up of a potential alternative therapy in these patients. We found that the expression of *CDK4* in CTCs after 1 cycle of therapy predicts an early progression. Interestingly, there is a general decrease in *CDK4* expression from 57.8 to 31.5% of the patients in response to docetaxel.

One important limitation of this study is the small number of patients enrolled. However, the serially sampling at different time points to characterize CTCs before and after chemotherapy and the chosen approach for CTCs isolation and normalization represent a strength of this analysis. In addition, at clinical progression only 13 samples were available for gene expression analysis, which hindered the obtaining of more conclusive results regarding docetaxel-resistant CTCs phenotype. Despite the small number of patients included in this study, gene expression analysis provides predictive biomarkers with better accuracy than CTCs counts or PSA levels. In the future, we aim to validate this data in a prospective study with a different and wider cohort of patients. Having additional sampling points before progression, in vitro experimental assays and an RNA-sequencing approach could help to understand the mechanisms underlying resistance, and is something to also explore in the future.

## Conclusions

This study takes novelty to the field and points to different predictive biomarkers depending on the time point of the disease, bringing out the molecular changes that have taken place throughout the time. Hence, we have reported for the first time *ZEB1* and *MYCL* in CTCs of mCRPC as potential biomarkers of resistance or response to taxanes respectively. In addition, this study supports the analysis of several markers together and not just a single representative for each phenotypic feature. We clearly evidenced that CTCs analysis allows the gathering of useful information even before the standard clinical evaluation (CT-Scan).

## Electronic supplementary material

Below is the link to the electronic supplementary material.

Electronic supplementary material 1 (DOCX 559 kb)

## Abbreviations

ADT: Androgen deprivation therapy
AR: Androgen receptors
CRPC: Castration-resistant PC
CSC: Cancer stem cell
CTC: Circulating tumour cell
EMT: Epithelial-mesenchymal transition
LB: Liquid biopsy
mCRPC: Metastatic CRPC
MET: Mesenchymal-epithelial transition
mPC: Metastatic PC
OS: Overall survival
PBMCs: Peripheral blood mononuclear cells
PC: Prostate cancer
PFS: Progression-free survival
PSA: Prostate-specific antigen
V1: Visit 1
V2: Visit 2
V3: Visit 3

## References

[1] Rawla P (2019). Epidemiology of prostate cancer. World J Oncol.

[2] Climent MÁ, León-Mateos L, González del Alba A (2014). Updated recommendations from the Spanish Oncology Genitourinary Group for the treatment of patients with metastatic castration-resistant prostate cancer. Crit Rev Oncol Hematol.

[3] Lonergan P, Tindall D (2011). Androgen receptor signaling in prostate cancer development and progression. J Carcinog.

[4] Lee CH, Kantoff P (2019). Treatment of metastatic prostate cancer in 2018. JAMA Oncol.

[5] Negoita S, Feuer EJ, Mariotto A (2018). Annual Report to the Nation on the Status of Cancer, part II: recent changes in prostate cancer trends and disease characteristics. Cancer.

[6] Scher HI, Morris MJ, Larson S, Heller G (2013). Validation and clinical utility of prostate cancer biomarkers. Nat Rev Clin Oncol.

[7] Pantel K, Hille C, Scher HI (2019). Circulating tumor cells in prostate cancer: from discovery to clinical utility. Clin Chem.

[8] Boerrigter E, Groen LN, Van Erp NP (2020). Clinical utility of emerging biomarkers in prostate cancer liquid biopsies. Expert Rev Mol Diagn.

[9] Scher HI, Jia X, de Bono JS (2009). Circulating tumour cells as prognostic markers in progressive, castration-resistant prostate cancer: a reanalysis of IMMC38 trial data. Lancet Oncol.

[10] De Bono JS, Scher HI, Montgomery RB (2008). Circulating tumor cells predict survival benefit from treatment in metastatic castration-resistant prostate cancer. Clin Cancer Res.

[11] Scher HI, Heller G, Molina A (2015). Circulating tumor cell biomarker panel as an individual-level surrogate for survival in metastatic castration-resistant prostate cancer. J Clin Oncol.

[12] Goldkorn A, Ely B, Quinn DI (2014). Circulating tumor cell counts are prognostic of overall survival in SWOG S0421: a phase III trial of docetaxel with or without atrasentan for metastatic castration-resistant prostate cancer. J Clin Oncol.

[13] Allard WJ, Matera J, Miller MC (2004). Tumor cells circulate in the peripheral blood of all major carcinomas but not in healthy subjects or patients with nonmalignant diseases. Clin Cancer Res.

[14] Leon-Mateos L, Elena C, Alicia A (2017). Improving circulating tumor cells enumeration and characterization to predict outcome in first line chemotherapy mCRPC patients. Oncotarget.

[15] Heller G, McCormack R, Kheoh T (2018). Circulating tumor cell number as a response measure of prolonged survival for metastatic castration-resistant prostate cancer: a comparison with prostate-specific antigen across five randomized phase III clinical trials. J Clin Oncol.

[16] Sharp A, Coleman I, Yuan W (2019). Androgen receptor splice variant-7 expression emerges with castration resistance in prostate cancer. J Clin Invest.

[17] Antonarakis ES, Lu C, Wang H (2014). AR-V7 and resistance to enzalutamide and abiraterone in prostate cancer. N Engl J Med.

[18] Antonarakis ES, Lu C, Luber B (2017). Clinical significance of androgen receptor splice variant-7 mRNA detection in circulating tumor cells of men with metastatic castration-resistant prostate cancer treated with first & second-line Abiraterone & Enzalutamide. J Clin Oncol.

[19] Markou A, Lazaridou M, Paraskevopoulos P (2018). Multiplex gene expression profiling of in vivo isolated circulating tumor cells in high-risk prostate cancer patients. Clin Chem.

[20] Josefsson A, Larsson K, Månsson M (2018). Circulating tumor cells mirror bone metastatic phenotype in prostate cancer. Oncotarget.

[21] Singhal U, Wang Y, Henderson J (2018). Multigene profiling of CTCs in mCRPC identifies a clinically relevant prognostic signature. Mol Cancer Res.

[22] Pereira-Veiga T, Martínez-Fernández M, Abuin C (2019). CTCs expression profiling for advanced breast cancer monitoring. Cancers (Basel).

[23] Cbioportal.org. (2020)

[24] Cerami E, Gao J, Dogrusoz U (2012). The cBio cancer genomics portal: an open platform for exploring multidimensional cancer genomics data. Cancer Discov.

[25] Gao J, Aksoy BA, Dogrusoz U (2013). Integrative analysis of complex cancer genomics and clinical profiles using the cBioPortal. Sci Signal.

[26] Li B, Dewey CN (2011). RSEM: Accurate transcript quantification from RNA-Seq data with or without a reference genome. BMC Bioinformatics.

[27] Amato RJ, Melnikova V, Zhang Y (2013). Epithelial cell adhesion molecule-positive circulating tumor cells as predictive biomarker in patients with prostate cancer. Urology.

[28] Onstenk W, Sieuwerts AM, Kraan J (2015). Efficacy of cabazitaxel in castration-resistant prostate cancer is independent of the presence of AR-V7 in Circulating tumor cells. Eur Urol.

[29] Klotz L, Teahan S (2006) Current role of PSA kinetics in the management of patients with prostate cancer. In: European urology, supplements, pp 472–478

[30] Scher HI, Graf RP, Schreiber NA (2017). Phenotypic heterogeneity of circulating tumor cells informs clinical decisions between AR signaling inhibitors and taxanes in metastatic prostate cancer. Cancer Res.

[31] Abreu M, Cabezas-Sainz P, Pereira-Veiga T (2020). Looking for a better characterization of triple-negative breast cancer by means of circulating tumor cells. J Clin Med.

[32] Markiewicz A, Topa J, Nagel A (2019). Spectrum of epithelial-mesenchymal transition phenotypes in circulating tumour cells from early breast cancer patients. Cancers (Basel).

[33] Bonk S, Kluth M, Hube-Magg C (2019). Prognostic and diagnostic role of PSA immunohistochemistry: a tissue microarray study on 21,000 normal and cancerous tissues. Oncotarget.

[34] Morrison GJ, Cunha AT, Jojo N (2020). Cancer transcriptomic profiling from rapidly enriched circulating tumor cells. Int J Cancer.

[35] de Wit S, Manicone M, Rossi E (2018). EpCAM(high) and EpCAM(low) circulating tumor cells in metastatic prostate and breast cancer patients. Oncotarget.

[36] Chang L, Graham PH, Hao J (2014). Emerging roles of radioresistance in prostate cancer metastasis and radiation therapy. Cancer Metastasis Rev.

[37] Topham C, Tighe A, Ly P (2015). MYC is a major determinant of mitotic cell fate. Cancer Cell.

[38] Koh CM, Bieberich CJ, Dang CV (2010). MYC and prostate cancer. Genes Cancer.

[39] Aaltonen KE, Novosadova V, Bendahl P-O (2017). Molecular characterization of circulating tumor cells from patients with metastatic breast cancer reflects evolutionary changes in gene expression under the pressure of systemic therapy. Oncotarget.

[40] Nakagawa M, Takizawa N, Narita M (2010). Promotion of direct reprogramming by transformation-deficient Myc. Proc Natl Acad Sci.

[41] Boutros PC, Fraser M, Harding NJ (2015). Spatial genomic heterogeneity within localized, multifocal prostate cancer. Nat Genet.

[42] Jie XX, Zhang XY, Xu CJ (2017). Epithelial-to-mesenchymal transition, circulating tumor cells and cancer metastasis: mechanisms and clinical applications. Oncotarget.

[43] Hanrahan K, O’Neill A, Prencipe M (2017). The role of epithelial-mesenchymal transition drivers ZEB1 and ZEB2 in mediating docetaxel-resistant prostate cancer. Mol Oncol.

[44] Hugo H, Ackland ML, Blick T (2007). Epithelial - Mesenchymal and mesenchymal - Epithelial transitions in carcinoma progression. J Cell Physiol.

[45] Wade CA, Kyprianou N (2018). Profiling prostate cancer therapeutic resistance. Int J Mol Sci.

[46] Figiel S, Vasseur C, Bruyere F (2017). Clinical significance of epithelial-mesenchymal transition markers in prostate cancer. Hum Pathol.

[47] Stemmler MP, Eccles RL, Brabletz S, Brabletz T (2019). Non-redundant functions of EMT transcription factors. Nat Cell Biol.

[48] Fazilaty H, Rago L, Kass Youssef K (2019). A gene regulatory network to control EMT programs in development and disease. Nat Commun.

[49] Brabletz T, Kalluri R, Nieto MA, Weinberg RA (2018). EMT in cancer. Nat Rev Cancer.

[50] Putzke AP, Ventura AP, Bailey AM (2011). Metastatic progression of prostate cancer and E-cadherin: regulation by ZEB1 and Src family kinases. Am J Pathol.

[51] Padmanaban V, Krol I, Suhail Y (2019). E-cadherin is required for metastasis in multiple models of breast cancer. Nature.

[52] Berger A, Brady NJ, Bareja R (2019). N-Myc-mediated epigenetic reprogramming drives lineage plasticity in advanced prostate cancer. J Clin Invest.

[53] Gulappa T, Reddy RS, Suman S (2013). Molecular interplay between cdk4 and p21 dictates G0/G1 cell cycle arrest in prostate cancer cells. Cancer Lett.

[54] Lynce F, Shajahan-Haq AN, Swain SM (2018). CDK4/6 inhibitors in breast cancer therapy: current practice and future opportunities. Pharmacol Ther.

