# Supplementary material for: Longitudinal CTCs gene expression analysis on metastatic castration-resistant prostate cancer patients treated with docetaxel reveals new potential prognosis markers
Source: Clin Exp Metastasis. 2021 Feb 26;38(2):239–51. doi: 10.1007/s10585-021-10075-1 (PMC7987626; doi:10.1007/s10585-021-10075-1)
Supplement: Supplementary file 1 — Electronic supplementary material 1 (DOCX 559 kb) [file 10585_2021_10075_MOESM1_ESM.docx]

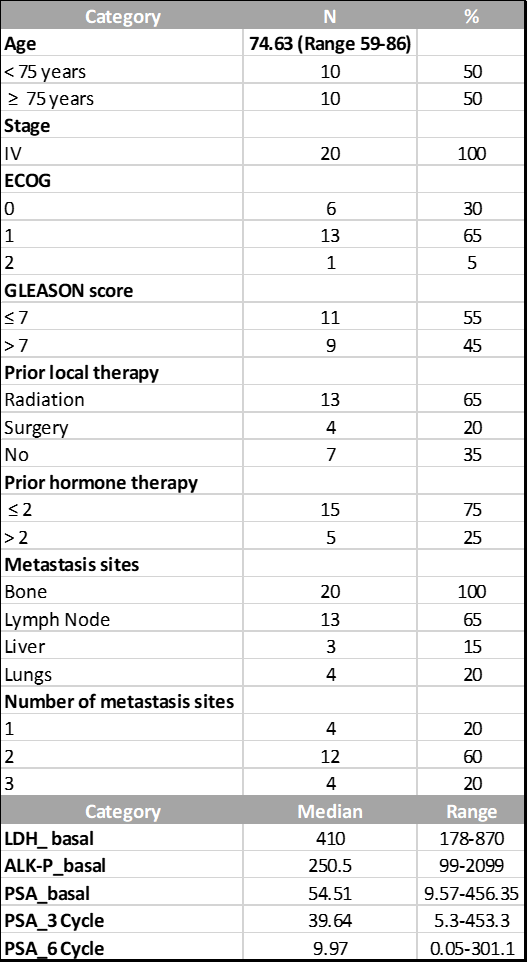


Table S1.Clinic-pathologic characteristics of the cohort of patients with mCRPC (n=20). Median values of LDH, ALK-P and PSA were measured in ng/mL.


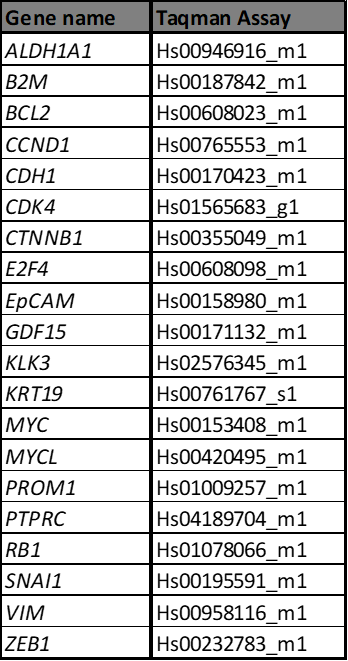


Table S2. List of Taqman probes used for qRT-PCR assay.


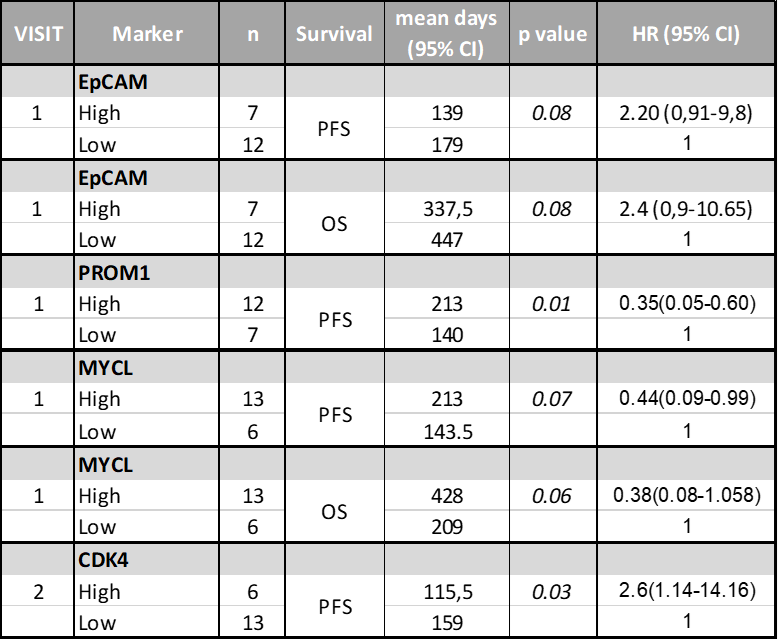

**Table S3.** Prognostic value of CTCs gene expression levels in mCRPC patients of the indicated markers. P-values were calculated using the log-rank test.


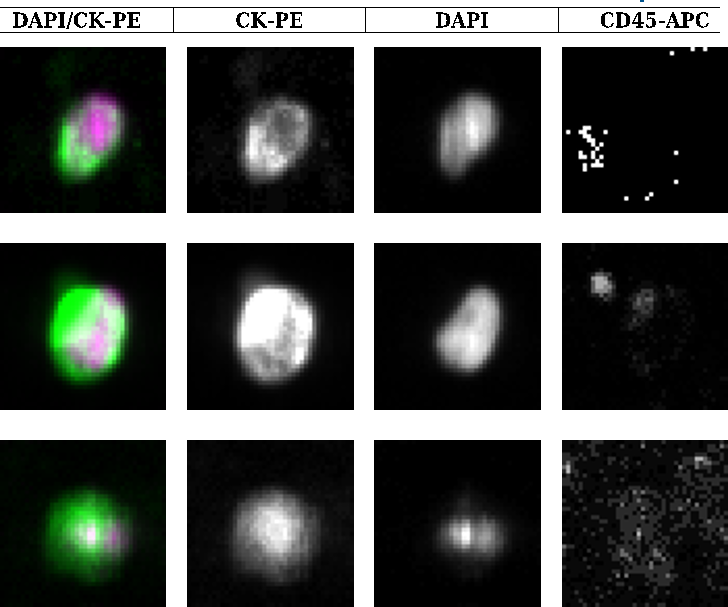


**Figure S1**. Representative CTCs images from CellSearch®. In the merge, CTCs showing CK 8, 18 and/or 19 (in green), DAPI+ (in pink) and absence of CD45 staining. Using the CellSearch® system, CTCs are enriched based on EpCAM+ expression, representing the epithelial or mixed subpopulation.


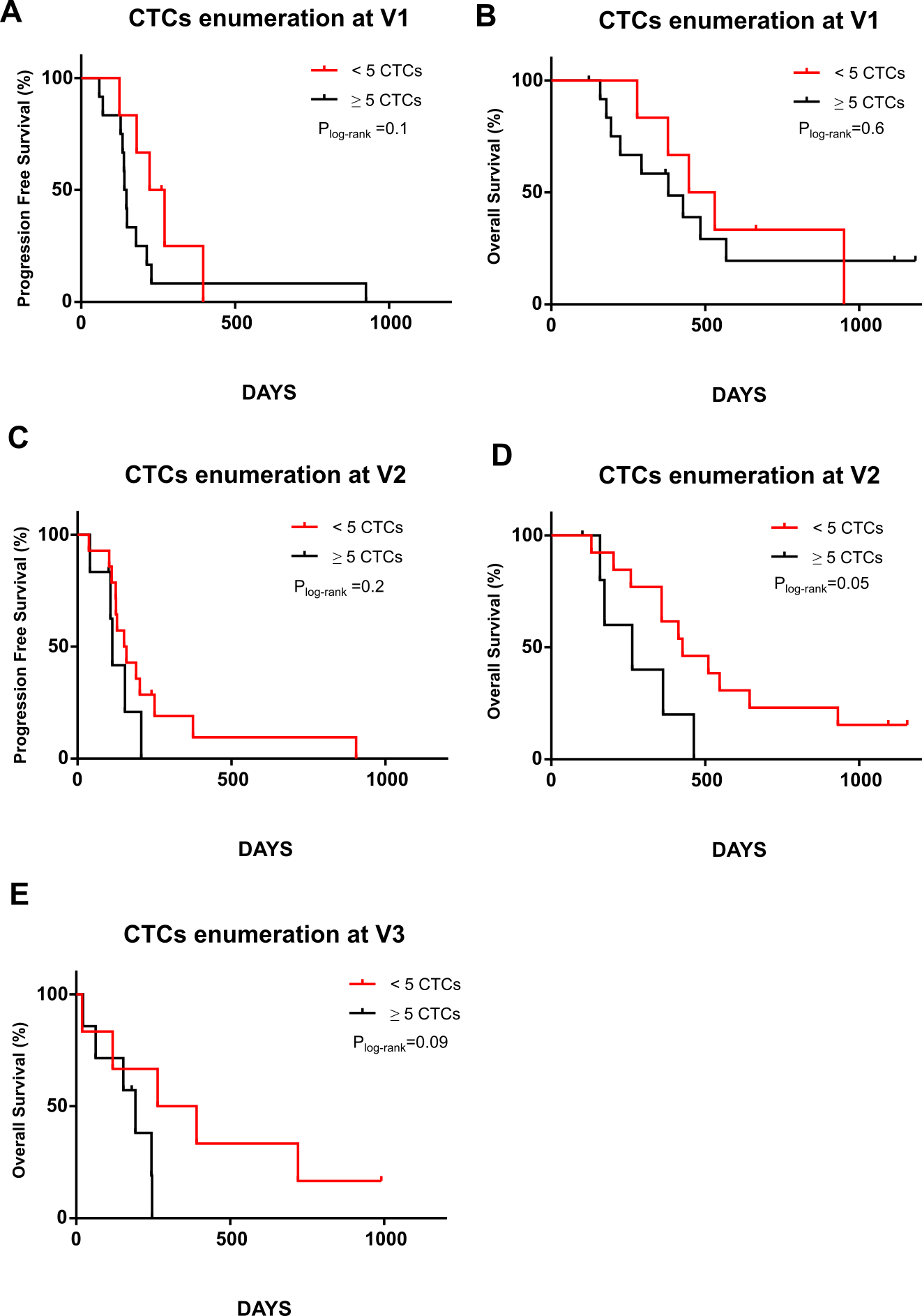


**Figure S2.** Kaplan–Meier plots for PFS and OS according to the CTCs count (low risk: < 5 CTCs, high risk: ≥ 5 CTCs), at the diagnosis of metastasis (V1: A, B), after one cycle of docetaxel (V2: C, D) and at the clinical progression (V3: E). P-values were calculated using the log-rank test.
